# Supplementary material for: A fault-intrusion-tolerant system and deadline-aware algorithm for scheduling scientific workflow in the cloud
Source: PeerJ Comput Sci. 2021 Nov 2;7:e747. doi: 10.7717/peerj-cs.747 (PMC8576554; doi:10.7717/peerj-cs.747)
Supplement: Supplemental Information 6 [file peerj-cs-07-747-s006.zip › Myproject1.1/docs/index-files/index-2.html]

B-Index


JavaScript is disabled on your browser.


- Overview
- Package
- Class
- Use
- Tree
- Deprecated
- Index
- Help

- Prev Letter
- Next Letter

- Frames
- No Frames

- All Classes

A B C D E F G H I J L M N O P Q R S T U V W Z 


## B

BagofTaskVm - Variable in class org.cloudbus.cloudsim.network.datacenter.Switch


BalancedClustering - Class in org.workflowsim.clustering.balancing
:   BalancedClustering is a clustering method that aims balancing task runtime
    and data dependency.

BalancedClustering(int) - Constructor for class org.workflowsim.clustering.balancing.BalancedClustering
:   Initialize a BalancedClustering method

BalancingMethod - Class in org.workflowsim.clustering.balancing.methods
:   BlancingMethod is the super class of all balancing methods

BalancingMethod(Map, Map, int) - Constructor for class org.workflowsim.clustering.balancing.methods.BalancingMethod
:   Initialize a BalancingMethod

BalancingMetric - Interface in org.workflowsim.clustering.balancing.metrics
:   Every balancing metric should implement this interface

bandwidth - Variable in class org.cloudbus.cloudsim.network.datacenter.NetworkHost


BandWidthAggRoot - Static variable in class org.cloudbus.cloudsim.network.datacenter.NetworkConstants


BandWidthEdgeAgg - Static variable in class org.cloudbus.cloudsim.network.datacenter.NetworkConstants


BandWidthEdgeHost - Static variable in class org.cloudbus.cloudsim.network.datacenter.NetworkConstants


BASE - Static variable in class org.cloudbus.cloudsim.network.datacenter.NetworkConstants


BASE - Static variable in class org.workflowsim.utils.Parameters


BasePlanner - Class in org.workflowsim.planning
:   The base planner has implemented the basic features.

BasePlanner() - Constructor for class org.workflowsim.planning.BasePlanner
:   Initialize a BaseScheduler

BaseScheduler - Class in org.workflowsim.scheduler
:   The base scheduler has implemented the basic features.

BaseScheduler() - Constructor for class org.workflowsim.scheduler.BaseScheduler
:   Initialize a BaseScheduler

BasicClustering - Class in org.workflowsim.clustering
:   The default clustering does no clustering at all, just map a task to a job

BasicClustering() - Constructor for class org.workflowsim.clustering.BasicClustering
:   Initialize a BasicClustering object

bindCloudletToVm(int, int) - Method in class org.cloudbus.cloudsim.DatacenterBroker
:   Specifies that a given cloudlet must run in a specific virtual machine.

bindSchedulerDatacenter(int, int) - Method in class org.workflowsim.WorkflowEngine
:   Binds a scheduler with a datacenter.

bindSchedulerDatacenter(int) - Method in class org.workflowsim.WorkflowEngine
:   Binds a datacenter to the default scheduler (id=0)

bindSchedulerDatacenter(int) - Method in class org.workflowsim.WorkflowScheduler
:   Binds this scheduler to a datacenter

BlockClustering - Class in org.workflowsim.clustering
:   BlockClustering groups tasks in both horizontal and vertical direction

BlockClustering(int, int) - Constructor for class org.workflowsim.clustering.BlockClustering
:   Initialize a BlockClustering object

broker - Static variable in class org.cloudbus.cloudsim.examples.power.RunnerAbstract
:   The broker.

buildNetworkTopology(String) - Static method in class org.cloudbus.cloudsim.NetworkTopology
:   Creates the network topology if file exists and if file can be succesfully parsed.

BUSY - Static variable in class org.cloudbus.cloudsim.Pe
:   Denotes Pe is allocated and hence busy in processing Cloudlet.

bwMatrix - Static variable in class org.cloudbus.cloudsim.NetworkTopology


BwProvisioner - Class in org.cloudbus.cloudsim.provisioners
:   BwProvisioner is an abstract class that represents the provisioning policy of bandwidth to
    virtual machines inside a Host.

BwProvisioner(long) - Constructor for class org.cloudbus.cloudsim.provisioners.BwProvisioner
:   Creates the new BwProvisioner.

BwProvisionerSimple - Class in org.cloudbus.cloudsim.provisioners
:   BwProvisionerSimple is a class that implements a simple best effort allocation policy: if there
    is bw available to request, it allocates; otherwise, it fails.

BwProvisionerSimple(long) - Constructor for class org.cloudbus.cloudsim.provisioners.BwProvisionerSimple
:   Instantiates a new bw provisioner simple.

A B C D E F G H I J L M N O P Q R S T U V W Z

- Overview
- Package
- Class
- Use
- Tree
- Deprecated
- Index
- Help

- Prev Letter
- Next Letter

- Frames
- No Frames

- All Classes
